# Mitochondria’s Role in Skin Ageing

**DOI:** 10.3390/biology8020029

**Published:** 2019-05-11

**Authors:** Roisin Stout, Mark Birch-Machin

**Affiliations:** Dermatological Sciences, Institute of Cellular Medicine, Medical School, Newcastle University, Newcastle upon Tyne NE2 4HH, UK; r.stout2@newcastle.ac.uk

**Keywords:** mitochondria, skin, ageing, reactive oxygen species, photoageing

## Abstract

Skin ageing is the result of a loss of cellular function, which can be further accelerated by external factors. Mitochondria have important roles in skin function, and mitochondrial damage has been found to accumulate with age in skin cells, but also in response to solar light and pollution. There is increasing evidence that mitochondrial dysfunction and oxidative stress are key features in all ageing tissues, including skin. This is directly linked to skin ageing phenotypes: wrinkle formation, hair greying and loss, uneven pigmentation and decreased wound healing. The loss of barrier function during skin ageing increases susceptibility to infection and affects wound healing. Therefore, an understanding of the mechanisms involved is important clinically and also for the development of antiageing skin care products.

## 1. Skin Structure

Skin is the largest organ of the human body and made up of three distinct layers: the epidermis, the dermis and subcutaneous fat. It functions as a barrier against the environment, providing protection against microbes as well as fluid and temperature homeostasis. The epidermis is a thin layer of densely packed keratinised epithelial cells (keratinocytes) which contains no nerves or blood vessels and relies on the thick dermal layer underneath for metabolism. The dermis is the main living tissue in the skin, consisting of fibroblast cells in an extracellular matrix interspersed with sweat glands, hair follicles, muscle, capillaries and nerve endings. The epidermal basal layer is the innermost layer of the epidermis dispersed with melanocytes [1]. It separates the outer layers of the epidermis from the nutrient-providing papillary dermis and the thick supporting reticular dermis layer below, all of which rest on a layer of subcutaneous fat. With skin taking a large environmental insult, the keratinocytes undergo constant turnover by epidermal stem cells to replace damaged cells [2]. Melanocytes produce melanin, which is transported to keratinocytes to produce skin pigmentation and provide some solar protection. Structural integrity, repair and strength within the dermal layer of the skin are provided by type I collagen fibres, produced from procollagen bodies. Collagen regulation occurs through synthesis promotion by cytokine TGF-β, inhibition by transcription factor AP-1 and active degradation by the collagenase enzymes matrix metalloproteinases (MMPs) [3].

## 2. Mitochondria’s Role in Skin

Mitochondria play a vital role in the skin. While the energy requirement may not be as great as other organs, such as skeletal muscle, it is still integral for processes like cell signalling, wound healing, pigmentation, vasculature homeostasis and hair growth. They are critical in microbial defence; glycolysis and ATP production have been found to rapidly increase in response to *Staphylococcus aureus* infection on the skin, in response to hypoxia induced metabolic stress [4]. This implements mitochondrial reactive oxygen species (ROS) signalling in the defence against skin infection through hypoxia-inducible factor 1-alpha (HIF1α) activation and immune cell recruitment [5]. Mitochondrial function and ROS production aid the regulation of stem cell differentiation, and have further been specifically linked to epidermal homeostasis and hair follicle development [6]. Reactive oxygen species signalling via mitochondria is therefore specifically involved in skin structure and function.

In patients with genetic mitochondrial disease, skin manifestations are often neglected as the disease predominantly affects the neuromuscular system, due to its high energy requirements. Various abnormalities in mitochondrial function, such as mutations of mitochondrial repair genes and haem synthesis, have been directly linked to a multitude of skin aberrations [7]. Lipomas and pigmentation disorders are the most commonly recorded skin complaints in mitochondrial disease patients [7,8]. This is likely due to mitochondrial defects in brown fat [9] and the direct effect of mitochondrial function in pigment production, respectively. Skin complaints can therefore be directly linked to genetic mitochondrial dysfunction and through complex ROS signalling. However, the correlation between the skin and mitochondria should be approached with caution, many other skin disorders are a result of secondary factors in diseases which do not primarily affect the mitochondrion itself, such as skin blistering in epidermolysis bullosa simplex [10].

## 3. Hallmarks of Skin Ageing

Wrinkles are one of the first features thought of when considering facial appearance and ageing. Intrinsic ageing is the result of chronological, inevitable senescence of the skin cells which varies depending on ethnicity, hormones and the anatomical region of affected skin. Extrinsic skin ageing is a result of all the external factors that can induce skin ageing, such as lifestyle, smoking, UV exposure and the environment, which have a cumulative effect over time [11]. Fine lines, breakdown of the skin structure, reddening due to increased visible vasculature and a reduction of elasticity are the main clinical features of intrinsic ageing; extrinsic ageing produces much deeper wrinkles, dryer skin, spider veins and uneven pigmentation [12].

As skin is constantly defending against environmental insult, it is important to maintain its integrity: ageing skin has reduced wound healing capacity and increased water loss. This increases susceptibility to cuts and infection, and makes it more prone to irritation and dermatoses [13]. It is essential to maintain an adequate skin barrier and understand the mechanisms involved in its loss to protect against age related dysregulation.

## 4. Mitochondria and Ageing

The “Free Radical Theory of Ageing” was first proposed by Harman in the 1950s [14]. It states that mutations acquired in mitochondrial DNA (mtDNA) during life, both spontaneously and through stress, can disrupt cellular metabolism like oxidative phosphorylation in the mitochondria and ultimately increase ROS. This, in turn, results in the oxidation of cellular components including proteins, lipids, DNA and RNA, which creates a cycle of altered metabolism and further damage. Ultimately, this results in the subsequent decline of cellular function seen in ageing and degenerative diseases.

Since it was first proposed, there have been a multitude of studies attempting to corroborate this theory by analysing mtDNA damage in ageing. As of yet there is no consensus, but there is a correlation between mtDNA damage, increasing oxidative stress and ageing. In skin samples, an accumulation of mtDNA deletions has been found to not only increase with age, but also in sun exposed areas compared to protected areas [15]. In this study by Ray et al., epidermal skin had a significantly greater increase in mtDNA deletions with chronic sun exposure compared to dermal skin, which had no significant change in mtDNA deletion quantity to protected skin. This was higher than those found in normal ageing. Additionally, an increase in point mutations has been observed in aged human fibroblasts which suggest that there are different types of mtDNA damage that could contribute to skin ageing [16]. Mitochondrial mutations and deletions have also been found to increase with ageing in other tissues. An accumulation of somatic mtDNA point mutations has been observed in the noncoding region of muscle tissue [17,18], and an increase in the frequency of a common 4977 bp deletion has been observed with increasing age in both breast and brain tissue [19,20]. This specific deletion has been found to increase in an age dependent manner in whole human skin [21], however more recent studies suggests that this increase only correlates with sun exposure [22] and particularly in the dermis [23]. The T414G transversion mutation in the mtDNA promoter region has a strong correlation with dermal fibroblast ageing, which is significantly increased in UV exposed regions [24]. This mutation has been found in tissues affected by age-related conditions such as in the brain of Alzheimer’s patients [25], precancerous cells in the colon [26] and an age-dependent increase in this mutation has also been found in muscle tissue [27]. This demonstrates that mtDNA damage increases with age in a variety of tissues and UV stress can accelerate this damage in the skin. However, it has been difficult to determine whether these mutations are generated by or induce ROS production, which is the centre of many debates surrounding this theory [28,29].

UV-induced oxidative stress and its role in signalling during extrinsic ageing have been well documented in the skin [30], but oxidative stress can also have ageing effects elsewhere. Reducing calorie intake by 10 to 50% is believed to decrease metabolic stress and scavenge ROS. It has been shown to increase lifespan in many organisms, including yeast [31] and mice [32], and decrease ageing biomarkers in nonhuman primates [33], making it a good candidate as a model to test Harman’s theory. Altogether, tissue-specific increase in mitochondrial efficiency and the oxidative stress response [34] and consequential reduction in ROS and oxidative stress have been shown following CR; and while not all CR study models increased in lifespan, there is evidence to suggest improved ageing health [35]. The focus of these studies is primarily lifespan and effects on organs other than the skin. However, a report on CR in Rhesus monkeys presented with subjective increase in hair loss in the ad libitum control compared to the CR monkey [36], therefore CR and the resulting metabolic changes could also have an impact on the skin.

Skin changes with CR were further investigated by Forni et al. [37] in mice. Caloric restriction resulted in increased mitochondrial function in the dermis, but not the epidermis, and fur remodelling and impaired vasoconstriction relating to thermoregulation. This implicates mitochondria as energy providers for cold adaptation in the skin, which agrees with previous observations of mitochondrial uncoupling as a preventative mechanism to cold stress [38]. In terms of skin ageing, CR promotes epidermal thickening and increases hair follicle stem cell pool which could indicate a rejuvenation of the skin by preventing skin thinning and hair loss indicative of skin ageing. This is coupled by evidence that mice without mitochondrial matrix antioxidant superoxide dismutase SOD2, which converts superoxide anon to hydrogen peroxide for further metabolism, exhibit normal ageing phenotypes at a younger age, particularly cellular senescence in the skin [39]. Therefore, the reduced capacity to break down superoxide anion results in higher oxidative stress and accelerated skin ageing in this model.

Another important discovery in mice looked at the induction of mtDNA depletion by an amino acid substitution in POLG1. Mice with this mutation showed a significant reduction in all OXPHOS complex activities in the skin and phenotypical ageing symptoms such as hair greying and loss, curvature of the spine, reduced movement and wrinkling of the skin [40]. The skin and hair changes are attributed to deformities of the hair follicle, increased epidermal cell proliferation causing deep wrinkles and epidermal thickening. Skin is not often commented on when addressing symptoms of POLG mutations in humans, so a satisfactory comparison cannot yet be made.

So far, research has demonstrated an increase of mtDNA mutations in ageing and a link between lower metabolic stress and increased ageing health, but does not show cause and effect. The problem with these models is two-fold. Firstly, is the amount of mutant mtDNA present in the cell enough to cause a pathogenic effect and secondly, do the observed age-related mutations dysregulate the respiratory chain? This can be partially answered with an observed decrease in mitochondrial function and metabolism in ageing muscle tissue in parallel with an increasing mutation load with ageing [41,42]. This does not rule out other mechanisms of mitochondrial dysfunction unrelated to the observed mutations [43], nor does it prove that ROS is a confounding factor in the pathogenesis.

## 5. Pigmentation

Melanin pigment is formed in response to oxidation reactions in melanocytes. Pheomelanin is the yellow-red pigment associated with red head and freckles and eumelanin which is more prevalent in dark haired individuals, the ratio of these is regulated by the melanocortin 1 receptor (MCR1) gene which has been previously reviewed [44]. Pheomelanin has been shown to have higher prooxidant effects on the cell as it sequesters cysteine and glutathione antioxidant during synthesis, and the loss-of-function in MCR1 results in the inability to produce eumelanin in response to α-melanocyte-stimulating-hormone (α-MSH), the process of which produces some antioxidant properties [45]. In terms of skin ageing, loss of MCR1 function has been linked to increased perceived age by an average of two years and heterozygous variants without complete loss-of-function had an increased perceived age of one year on average [46]. It has been suggested that this could be the effect of oxidative effects of pheomelanin production or changes to fibroblast function in the absence of MCR1 activity. In relation to mitochondrial function, melanocytes pretreated with α-MSH have been shown to have a protective effect on mtDNA copy number in response to UVB light. This could infer that stimulation of eumelanin rather than pheomelanin could have photoprotective properties; however, no quantification pheomelanin/eumelanin was performed [47]. Links between mitochondrial function, mtDNA and pheomelanin/eumelanin ratio have not yet been formally researched, which could provide an insight into the mechanisms involved in skin ageing though oxidative stress.

Uneven pigmentation is one of the hallmarks of skin ageing. Mitochondria have even been implicated in the biosynthesis of melanin in melanocytes, required to create pigmentation in response to UV light. Prohibitin proteins found localised in the inner mitochondrial membrane were found to bind directly to melanogenin, a synthetic pigmentation promotor. Prohibitin silencing directly interfered with melanogenin activity and is thought to be involved in the regulation of rate limiting melanin enzyme tyrosinase [48]. In addition to this, mitochondria in melanocytes have been shown to interact with melanosomes, suggesting a functional role in melanosome biogenesis and therefore melanin production [49].

Melatonin is a hormone predominantly produced in the brain to regulate sleep, and its metabolism relies heavily on mitochondria and ROS signalling [50]. It exhibits antioxidant effects and can inhibit melanogenesis in animal models at high concentrations, which can help modulate coat colour [51]. Studies observing changes in human skin pigmentation with intake of oral melatonin found no effects over a 30-day period in patients with hyperpigmentation of varying causes [52], melanoma patients or a control group [53]. However, melatonin and its metabolites have been detected in human skin in vivo, and all were found to inhibit melanocyte proliferation and tyrosinase activity in vitro [54]. All of the above suggests that mitochondrial function is a modulator of skin pigmentation, and also that dysfunction could interfere with melanin production both directly and indirectly through excessive ROS signalling and melatonin production.

These findings correspond with the observation from individuals with vitiligo—a condition which results in areas of the skin with inactive melanocytes—of reduced energy production in cultured melanocytes with depleted skin pigmentation, compared to healthy melanocytes [55], and the increased presence of vitiligo in patients with genetic mitochondrial dysfunction [56].

## 6. Photoageing

Sun damage is a well-known cause of skin cancer and ageing, and photoageing is the process of chronic sun exposure leading to extrinsic skin ageing. Solar light is composed of ultraviolet radiation (UVR) (10–380 nm), visible light (380–780 nm) and infrared radiation (IR) (above 780 nm). UVR is the most notorious for causing skin damage, so protection against both UVA and UVB is found in most sunscreens. UVB radiation was once believed to be the only contributor to photoageing due to higher energy levels than UVA, but it is now proven that UVA is the key player, though each can affect skin in different ways. UVA makes up the majority of solar UVR but only affects DNA indirectly, whereas UVB radiation represents a small portion but causes direct DNA damage [57].

**Figure 1:**
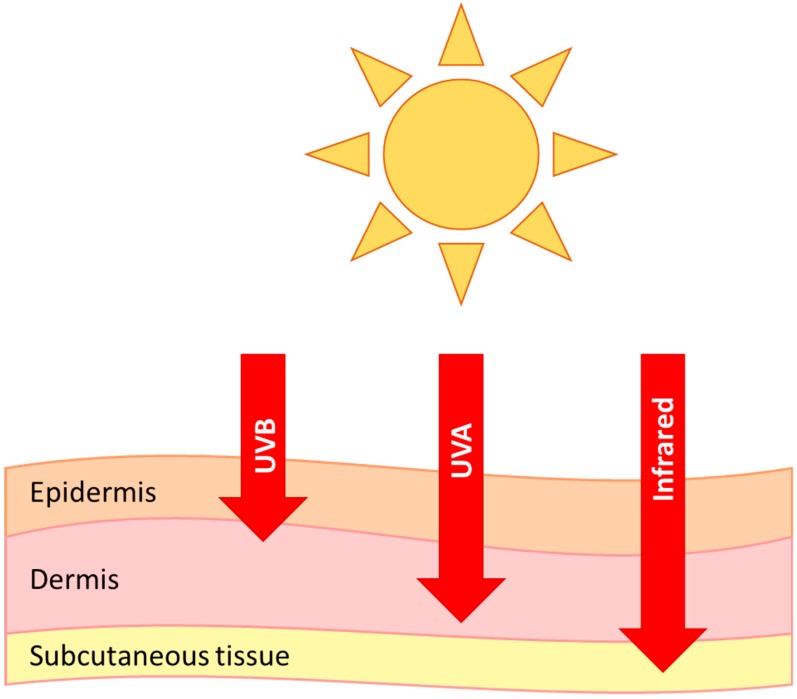
Penetration of UVB, UVA and infrared radiation into the skin.

The epidermis is the first line of defence against UVB damage and absorbs the majority of the radiation. The level of which depends on ethnicity, site of skin, hydration and many other factors [58]. Pigmentation from melanin plays a large part in the initial protection against UVR [59], and higher melanin has an inverse correlation with DNA lesions in humans [60] and this is also seen in other species, such as whales [61]. UVA, visible and infrared light can penetrate deeper into the skin than UVB (Figure 1), and it has been shown that dermal fibroblasts are in fact more susceptible to DNA damage from longer wavelengths of light (>300 nm) [62]. There is also evidence of visible and infrared light induced skin damage, and even a synergistic effect of all wavelengths [63].

Ageing is a natural process, and even without UV insult would occur over time due to the gradual shortening of telomere caps in nuclear DNA, loss-of-functionality in ageing and consequent cellular senescence [64]. This ageing damage profile has been found in a number of age related diseases including dementia and atherosclerosis [65,66]. It can also be induced in vitro in skin fibroblasts using UVA radiation [67] and in keratinocytes using UVB exposure [68], which further demonstrates a role for UVR in ageing. In addition to this, mtDNA damage is repeatedly seen in higher levels in photoexposed skin leading to an accumulation of cellular damage and a decrease in mitochondrial activity in the skin [24,69,70,71].

Although the precise mechanisms of photoageing are still being researched, collagen, mtDNA damage and increased ROS production are all key features. Inflammation induces the breakdown of matrix proteins to help the recruitment and migration of immune cells, therefore chronic inflammation can result in detrimental breakdown of tissues [72]. Inflammation due to UVA and UVB exposure has been shown to induce the enzymatic activity of MMPs in the dermis and epidermis, respectively, through ROS signalling and the activation of transcription factor AP-1 and NFκB [73,74,75,76]. AP-1 production reduces synthesis of procollagen type I and III, thereby disrupting the formation of new collagen at the same time as degrading it [77]. This is supported by the presence of ultrastructural changes and degradation of collagen fibre bundles from the dermis in photoexposed areas of human skin and UV irradiated mouse models [78]. Infrared radiation can also induce MMPs in dermal fibroblasts [79]. Therefore, sun exposure is directly linked to the active breakdown of collagen and the reduction of collagen synthesis through ROS signalling, leading to the formation of deep wrinkles and extrinsic skin ageing. Hypothetically, the mtDNA damage observed in photoexposed areas could cause dysregulation of OXPHOS and an increase in ROS production, as theorised in the ‘Free Radical Theory of Ageing’, and this could then lead to accelerated skin ageing.

The process of photodegradation of melanin results in singlet oxygen production, which has been shown in synthetic models [80], and UVA irradiation of pheomelanin exhibited conformational changes in catalase antioxidant which was attenuated with a singlet oxygen quencher [81]. In humans, a six-fold increase in photoageing was observed in those with homozygous loss of MC1R gene variants compared to those with wild type gene [82]. This all suggests that photodegradation of melanin could also be an important mechanism in the oxidative stress and mtDNA damage exhibited in photoexposed skin.

## 7. Pollution

With climate change a major danger to human health, research into the effects of air pollution has received growing attention. Outdoor pollutants predominantly originate from vehicle emissions, combustion of fossil fuels and industrial processes. Small particulate matter (PM_2.5_) and ozone has been shown to increase DNA and protein damage through ROS and oxidative stress in vitro and in mouse models [83,84], and have a positive association with skin ageing though pigmentation spots and skin wrinkling in Chinese [85] and German populations [86]. Activation of the aryl hydrocarbon receptor (AhR) is key in modulating the effects of pollution. Its activation by ligands, including ozone and PM_2.5_ [87,88], increases the expression of cytochrome P450, which can metabolise polycyclic aromatic hydrocarbons into carcinogenic substances capable of inducing DNA damage. These mechanisms have been further reviewed elsewhere [12,89,90].

Lentigines are hyperpigmented lesions in the skin as a consequence of excess melanocytes, which often become more abundant during ageing. Solar lentigines are associated with the level of sun exposure of the skin [91], and are the product of excess melanin production. Traffic-associated particles and soot have also been associated with the formation of these pigment spots on the forehead and cheeks [86,92], suggesting a common mechanism between UVR and pollution stressors. It has been suggested that one factor in this could be due to the age-dependent decline of mitochondrial complex II: succinate dehydrogenase [93]. Complex II is entirely transcribed by nuclear DNA and has been shown to decrease it’s activity in comparison to complex I and IV in an age dependent manner in senescent cells, predominantly in the epidermis [69]. Inducing hyperpigmentation in melanoma cells results in the inhibition of complex II activity and an initial increase in superoxide ROS formation, and increasing melanin concentration was capable of attenuating further formation [94]. Therefore, lentigine formation could be a protective mechanism against the increase ROS production from senescent skin cells, due to a decline in complex II function.

## 8. Hair Follicles

Hair loss and greying is another key observable ageing phenotype. Unfortunately, this is one ageing phenotype which is poorly understood and as yet cannot be prevented. Hair follicles are situated within the dermis, and melanocytes and keratinocytes are responsible for the colour and growth of hair; it is therefore important to mention hair within the context of skin and ageing. 

One theory of hair greying involves the acquisition of mitochondrial DNA damage with increased oxidative stress; the ‘common’ 4977 bp deletion has been discovered in higher levels in greying and unpigmented hair follicles (40% and 20%, respectively) than in pigmented (5%) [95]. Greying hair can be characterised by the gradual loss of melanocytes in the pigmentary units by apoptosis, until the hair follicle is unpigmented [96]. This pigmentary unit in greying hair was found to have the highest level of oxidative stress in melanocytes and a loss of ROS scavenging Bcl-2 activity. Bcl-2 is an inhibitor of the mitochondrial pathway of apoptosis, so the diminished expression can explain the increased apoptosis of melanocytes in response to cell stressors. These findings are in line with the grey hair phenotype of Bcl2−/− mice [97]. This details the mechanisms associated with mitochondrial function; but all of three theorised mechanisms, as reviewed by SK Jo et al. (2018) [98], point to a role of oxidative or genotoxic stress induced damage, which supports mitochondrial involvement.

Hair loss can occur though ageing, nutrient deficiency and temporary or permanent alopecia, all of which present differently. Like hair greying, hair loss is little understood. Mice with PolgA mutations leading to a faulty mtDNA repair mechanism, have an increased mtDNA mutation abundance and an accelerated ageing phenotype which exhibits a reduction in hair density [99]. Age-related hair loss in humans is predominantly androgenic with a higher prevalence in men [100]. Dermal papilla cells from balding regions have been found to express higher levels of senescence markers and have antioxidant superoxide dismutase expression localised in the nuclear region. This is in contrast to the cytoplasmic or mitochondrial localisation found in nonbalding counterparts [101]. This translocation of superoxide dismutase in response to oxidative stress in the cell is a protective mechanism as it is suggested to promote oxidative resistance and repair and function as a transcription factor [22].

Mice with mitochondrial transcription factor A (TFAM) knockdown, which causes mtDNA depletion and loss of electron transport chain (ETC) complexes, in the epidermis and hair follicle epithelium show a reduction in proliferation, increase in apoptosis and exhibit abnormalities in melanin production and function in the hair follicles [102]. ROS signalling from mitochondrial function is required for hair growth, as shown by high mitochondrial membrane potential and a burst of ROS production at the matrix epidermal cell and hair shaft interface in growing cultured bovine and human hair follicles [103]. These epithelial cells subsequently underwent mitochondrial de-polarisation as they converted to hair shaft matrix. Both these studies demonstrate critical mitochondrial function and ROS signalling in the morphogenesis of hair follicles and hair shaft elongation, and the importance of functional ETC in these processes.

Although there is a limited understanding of hair ageing, there is evidence to support mitochondrial involvement in both, predominantly through the increase of oxidative stress and a reduced capacity to cope.

## 9. Summary

Mitochondria are important for skin function and mtDNA mutations; functional decline is linked to skin ageing through stress-induced wrinkle formation, pigmentation and hair greying and loss. Many of the mechanisms contributing to skin ageing are not fully understood, and it is likely that mitochondrial function is part of a complex set of processes that lead to tissue functional decline and ageing. It is important scientifically to understand the mechanisms behind skin ageing to clinically develop prevention strategies, but it is also useful in industry to improve antiageing formulas and those targeted to mature skin.

## Abbreviations

UVA	Ultraviolet A
UVB	Ultraviolet B
IR	Infrared
MMPs	Matrix metalloproteinases
ROS	Reactive oxygen species
AP-1	activator protein-1
NFκB	Nuclear factor kappa-light-chain-enhancer of activated B cells
TGF-β	Transforming growth factor beta
PM_2.5_	Small particulate matter
AhR	Aryl hydrocarbon receptor
Bcl-2	B-cell lymphoma 2
ETC	Electron transport chain
